# Supplementary figures and images for: Bps polysaccharide of Bordetella pertussis resists antimicrobial peptides by functioning as a dual surface shield and decoy and converts Escherichia coli into a respiratory pathogen
Source: PLoS Pathog. 2022 Aug 15;18(8):e1010764. doi: 10.1371/journal.ppat.1010764 (PMC9410548; doi:10.1371/journal.ppat.1010764)

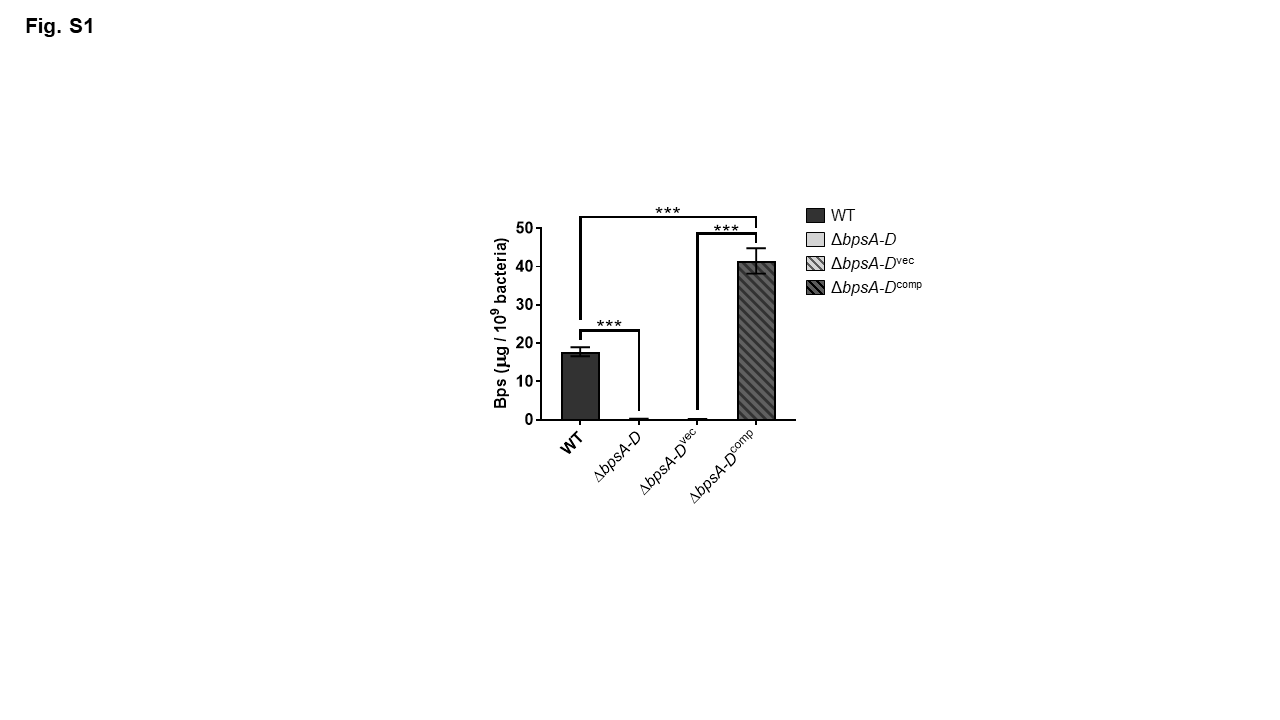

Supplement: S1 Fig — Quantitation of Bps from WT, ΔbpsA-D, ΔbpsA-Dvec, and ΔbpsA-Dcomp strains by ELISA. Each data point represents the mean and s.e.m. of n = 10 from one experiment and is representative of three independent experiments. Statistical differences were assessed by two-way ANOVA. ***, p<0.0005. (TIF) [file ppat.1010764.s001.tif]

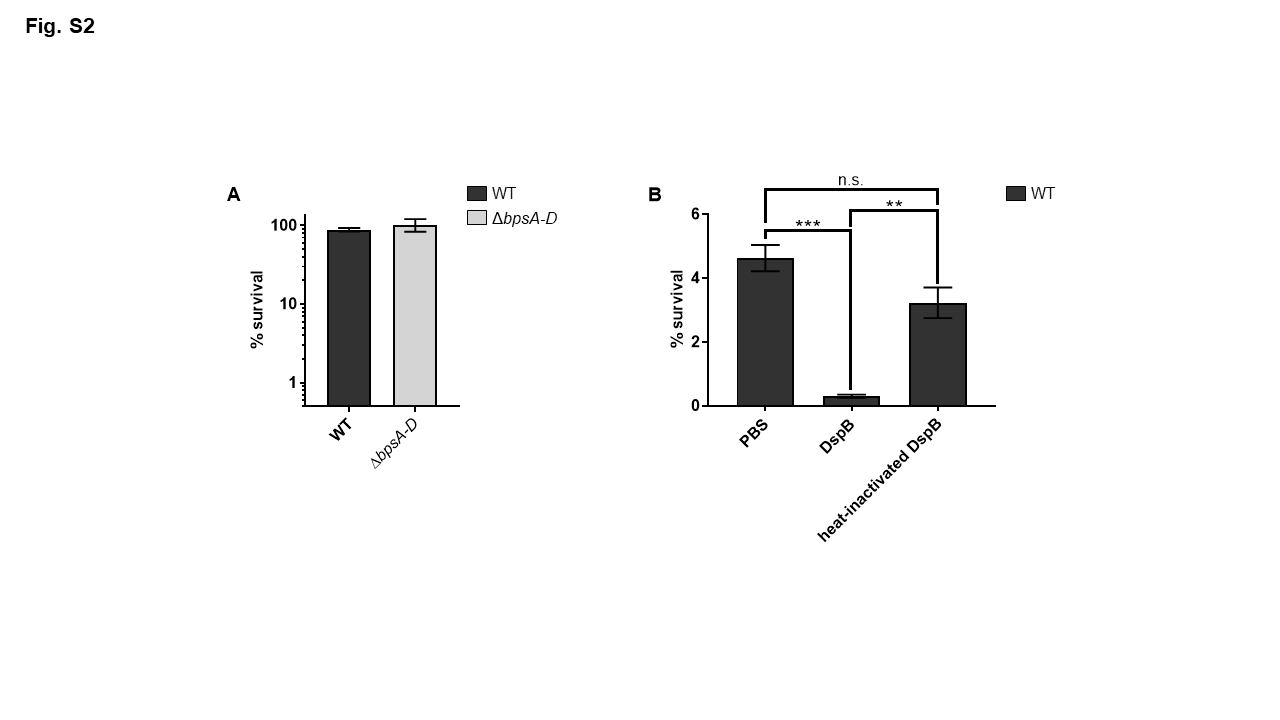

Supplement: S2 Fig — (a) Survival of WT and ΔbpsA-D strains after incubation for 2 hours with Dispersin B at 37°C. Each data point represents the mean and s.e.m. of triplicates from one experiment and is representative of two independent experiments. Statistical differences were assessed by unpaired two-tailed Student’s t test. (b) Survival of WT bacteria following treatment with PBS, 50 μg/ml Dispersin B, or 50 μg/ml heat-inactivated Dispersin B in the presence of .5 μg/ml LL-37. Dispersin B was inactivated by incubation at 56°C for 30 minutes. Each data point represents the mean and s.e.m. of triplicates from one experiment and is representative of two independent experiments. Statistical differences were assessed by one-way ANOVA. **, p<0.005; ***, p<0.0005. (TIF) [file ppat.1010764.s002.tif]

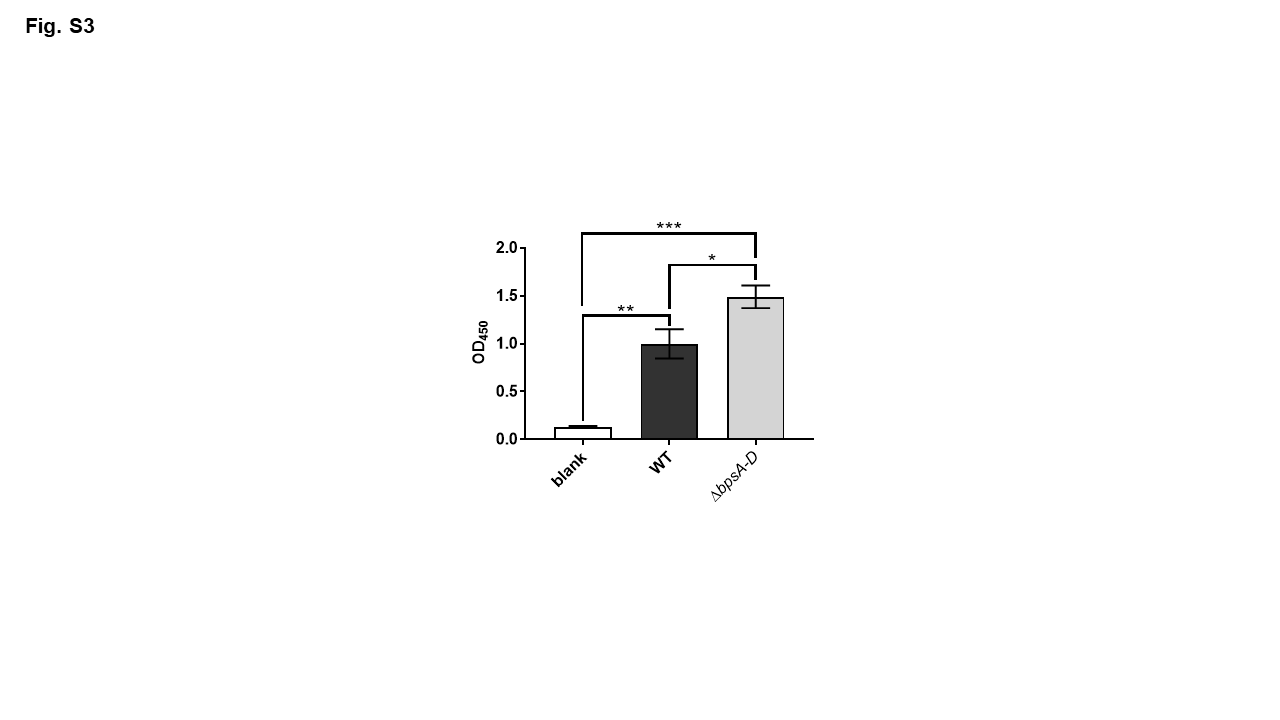

Supplement: S3 Fig — Binding of polymyxin B to WT and ΔbpsA-D strains was determined by ELISA utilizing a mouse monoclonal anti-polymyxin B antibody, as described in the Materials and Methods. Blank designates wells where PBS was added instead of bacterial cells. Each data point represents the mean and s.e.m. of triplicates from one of two experiments. Statistical differences were assessed by one-way ANOVA. *, p<0.05; **, p<0.005; ***, p<0.0005. (TIF) [file ppat.1010764.s003.tif]

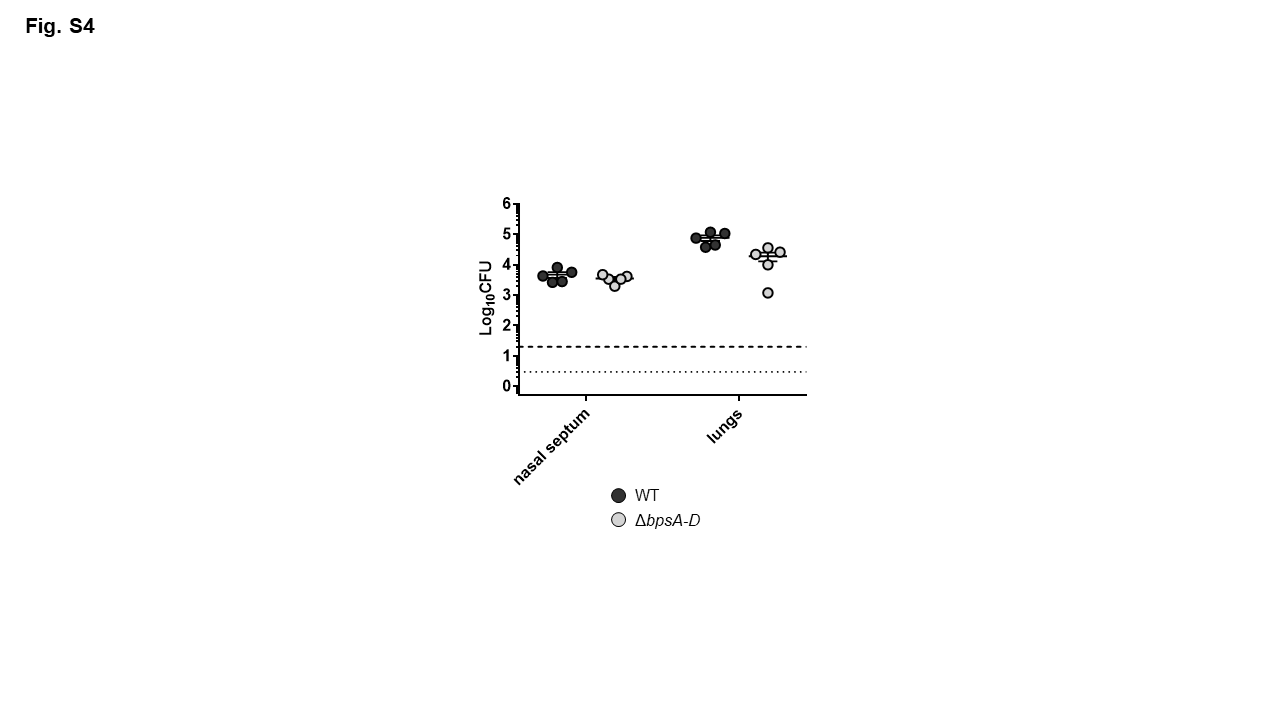

Supplement: S4 Fig — Bacterial CFUs recovered from the nasal septum and lungs of C57BL/6J mice 30 minutes after aerosol infection with co-culture of WT and ΔbpsA-D strain in a 1:1 ratio. Bars indicate the mean and s.e.m. of groups of five mice each. Data are representative of one of two independent experiments. Statistical differences were assessed by two-tailed Student’s t test for each organ. Dotted line represents the lower limit of detection for nasal septum, and dashed line represents the lower limit of detection for lungs. (TIF) [file ppat.1010764.s004.tif]

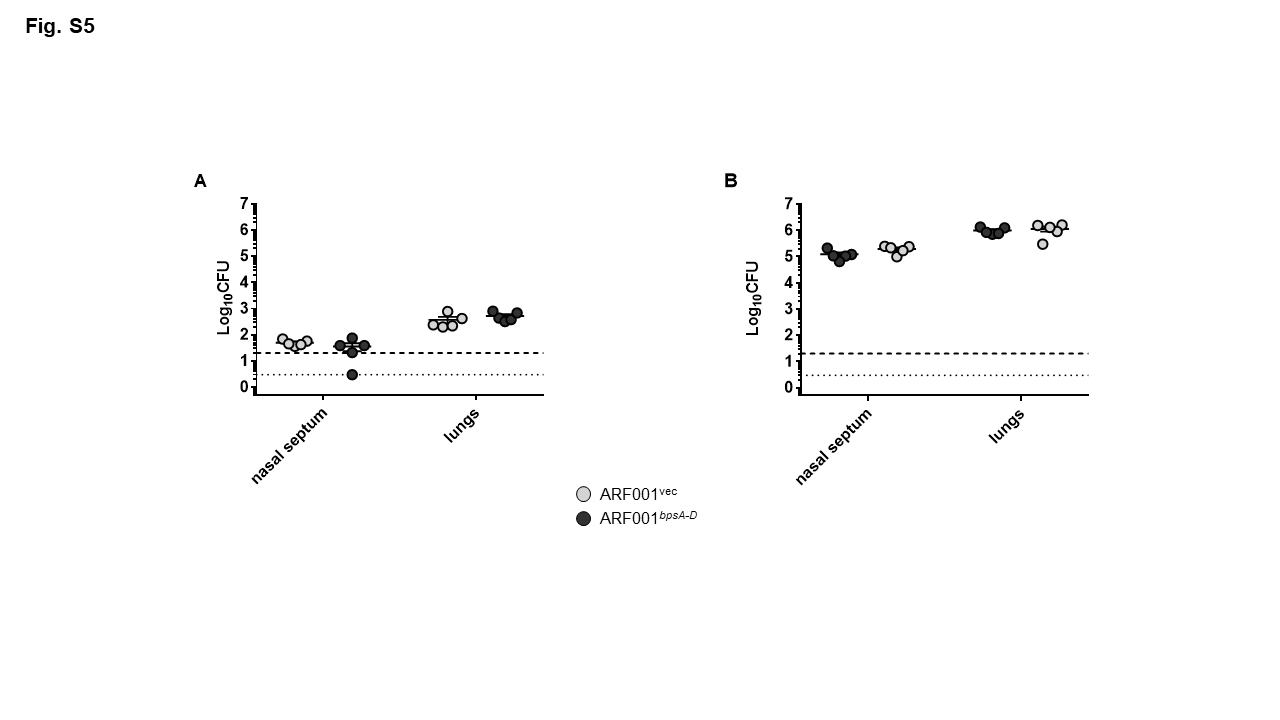

Supplement: S5 Fig — Bacterial CFUs recovered from the nasal septum and lungs 30 minutes after aerosol (a) or intranasal (b) challenge with either the ARF001vec or ARF001bpsA-D strains. Bars indicate the mean and s.e.m. of groups of five mice each. Data are representative of one of two independent experiments. Statistical differences were determined by unpaired two-tailed Student’s t test for each organ. Dotted line represents the lower limit of detection for nasal septum, and dashed line represents the lower limit of detection for lungs. (TIF) [file ppat.1010764.s005.tif]

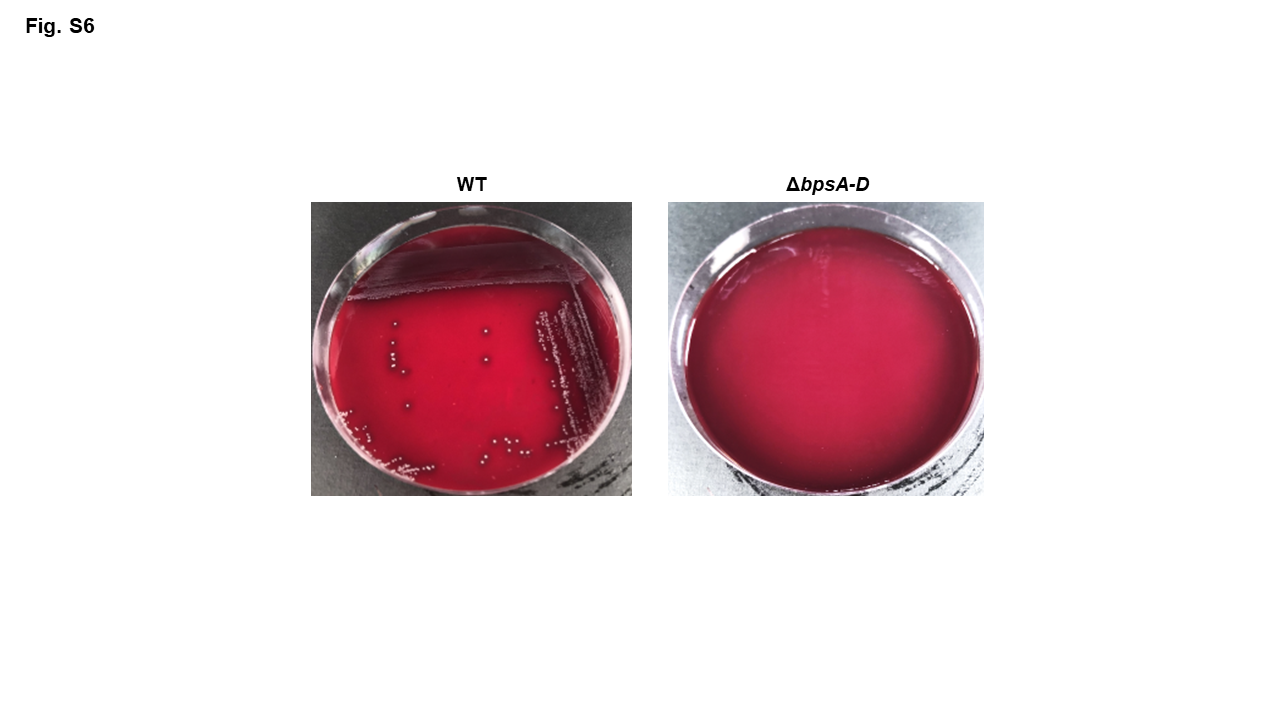

Supplement: S6 Fig — WT and ΔbpsA-D strains were streaked on BG agar supplemented with 10% defibrinated sheep blood and 20 μg/ml nalidixic acid. Plates were incubated at 37°C for four days. (TIF) [file ppat.1010764.s006.tif]
