# Supplementary material for: Bps polysaccharide of Bordetella pertussis resists antimicrobial peptides by functioning as a dual surface shield and decoy and converts Escherichia coli into a respiratory pathogen
Source: PLoS Pathog. 2022 Aug 15;18(8):e1010764. doi: 10.1371/journal.ppat.1010764 (PMC9410548; doi:10.1371/journal.ppat.1010764)
Supplement: S1 Table — (DOCX) [file ppat.1010764.s007.docx]

**S1 Table**

| **Strain or Plasmid** | **Characteristics** | **Reference or Source** |
| --- | --- | --- |
| **Strains** | | |
| ***B. pertussis*** | | |
| WT | Bp536 reference strain; Sm^R^, Nal^R^ | laboratory stock |
| Δ*bpsA-D* | Bp536 derivative containing an in-frame deletion of the *bpsA-D* locus; Sm^R^, Nal^S^ |  |
| Δ*bpsA-D*^vec^ | Δ*bpsA-D* strain containing the vector plasmid pBBR1MCS; Cm^R^ | [1] |
| Δ*bpsA-D*^comp^ | Δ*bpsA-D* strain containing the plasmid pMM11; Cm^R^ | [1] |
| ***E. coli*** | | |
| ARF001^vec^ | MG1655 *csrA*::*kan* strain containing a deletion in the *pgaA-D* locus and containing the plasmid pBBR1MCS; Cm^R^ | [2] |
| ARF001*^bpsA-D^* | MG1655 *csrA*::*kan* strain containing a deletion in the *pgaA-D* locus and containing the plasmid pMM11; Cm^R^ | [2] |
| **Plasmids** | | |
| pBBR1MCS | broad host-range vector plasmid; Cm^R^ | [3] |
| pMM11 | *bpsA-D* locus cloned into pBBR1MCS; Cm^R^ | [4] |

References:

1. Conover MS, Sloan GP, Love CF, Sukumar N, Deora R. The Bps polysaccharide of Bordetella pertussis promotes colonization and biofilm formation in the nose by functioning as an adhesin. Mol Microbiol. 2010;77(6):1439-55. Epub 2010/07/17. doi: MMI7297 [pii]

10.1111/j.1365-2958.2010.07297.x. PubMed PMID: 20633227.

2. Wang X, Preston JF, 3rd, Romeo T. The pgaABCD locus of Escherichia coli promotes the synthesis of a polysaccharide adhesin required for biofilm formation. J Bacteriol. 2004;186(9):2724-34. Epub 2004/04/20. PubMed PMID: 15090514.

3. Kovach ME, Phillips RW, Elzer PH, Roop RM, 2nd, Peterson KM. pBBR1MCS: a broad-host-range cloning vector. Biotechniques. 1994;16(5):800-2. Epub 1994/05/01. PubMed PMID: 8068328.

4. Parise G, Mishra M, Itoh Y, Romeo T, Deora R. Role of a putative polysaccharide locus in Bordetella biofilm development. J Bacteriol. 2007;189(3):750-60. Epub 2006/11/23. doi: JB.00953-06 [pii]

10.1128/JB.00953-06. PubMed PMID: 17114249.
