# Supplementary material for: Bps polysaccharide of Bordetella pertussis resists antimicrobial peptides by functioning as a dual surface shield and decoy and converts Escherichia coli into a respiratory pathogen
Source: PLoS Pathog. 2022 Aug 15;18(8):e1010764. doi: 10.1371/journal.ppat.1010764 (PMC9410548; doi:10.1371/journal.ppat.1010764)
Supplement: S2 Table — (DOCX) [file ppat.1010764.s008.docx]

**S2 Table**

|  |  | **Histopathological Scoring Parameters** |  |
| --- | --- | --- | --- |
|  | **Degree of Cellularity/Consolidation** | **Thickness of Alveolar Walls** |  |
| **0** | Cellular infiltrate/consolidation absent | Alveolar wall thickness within normal limits |  |
| **1** | Mild (< 25%) infiltration and consolidation | Mild: < 25% of alveolar walls thickened |  |
| **2** | Moderate (26-50%) infiltration and consolidation | Moderate: 26-50% of alveolar walls thickened |  |
| **3** | Marked (51-75%) infiltration and consolidation | Marked: 51-75% of alveolar walls thickened |  |
| **4** | Severe (> 75%) infiltration and consolidation | Severe: > 75% of alveolar walls thickened |  |
| **5** | Complete consolidation with no clear alveoli | All alveolar walls thickened, no walls within normal limits |  |
|  | **Alveolar/Interstitial Polymorphonuclear Cells (PMNs)** | **Intrabronchial PMNs** | **Alveolar Macrophages** |
| **0** | PMNs absent | PMNs absent | Absent |
| **1** | Mild: < 25% of alveoli/interstitium contain PMNs | Mild: < 25% of bronchi/bronchioles contain PMNs | Mild: < 25% of alveoli contain macrophages |
| **2** | Moderate: 26-50% of alveoli/interstitium contain PMNs | Moderate: 26-50% of bronchi/bronchioles contain PMNs | Moderate: 26-50% of alveoli contain macrophages |
| **3** | Marked: 51-75% of alveoli/interstitium contain PMNs | Marked: 51-75% of bronchi/bronchioles contain PMNs | Marked: 51-75% of alveoli contain macrophages |
| **4** | Severe: > 75% of alveoli/interstitium contain PMNs | Severe: > 75% of bronchi/bronchioles contain PMNs | Severe: > 75% of alveoli contain macrophages |
| **5** | All areas of examined lung sample contain PMNs | PMNs present in all evaluated airways | Macrophages present in all evaluated alveoli |
